# Failed internal fixation

**DOI:** 10.3109/17453674.2011.555373

**Published:** 2011-02-10

**Authors:** Praveen Mereddy

**Affiliations:** 9 St Brides Court, Ingleby Barwick, Stockton-on-Tees, TS17 5HF Cleveland, UKmpkr3@yahoo.com

*Sir*—We read this article (Zustin and Winter 2009) with great interest and we appreciate the great efforts the authors have put in to publish this case report and extensive review of literature. However, we would like to discuss some primary issues regarding the case that has been reported.

The authors made an excellent effort to salvage the prosthesis by internal fixation. However, this fixation method is not ideal even for a simple fractured neck of femur let alone periprosthetic fracture following hip resurfacing. It is well established that the more vertical the fracture neck of femur, higher is the incidence of non-union and avascular necrosis (Liporace et al. 2008)

It would be of some interest to review the pre-injury radiograph with the hip resurfacing in situ to rule out superior notching as this fracture pattern is usually associated with superior notching and trauma in a young patient with good quality of bone.

In a morphological study (Zustin et al. 2010c), osteonecrosis was the most frequent cause of fracture-related failures. The authors also suggested that intraoperative mechanical injury of the femoral neck such as notching and/or malpositioning of the femoral component might lead to changes in the loading pattern or in the resistance to fracture of the femoral neck and may result in both acute and chronic biomechanical femoral neck fractures.

We note the authors comments that the avascular necrosis was possibly after the second procedure from the histology findings. However, it is difficult to ascertain whether the avascular necrosis was the cause of the fracture or it was secondary to the trauma that possibly disrupted the blood supply from the minimally displaced fracture.
