# Supplementary material for: Failed internal fixation
Source: Acta Orthop. 2011 Feb 10;82(1):119–21. doi: 10.3109/17453674.2011.555373 (PMC3230009; doi:10.3109/17453674.2011.555373)
Supplement: Related texts

# Letter: Failed internal fixation

**Published:** 2011-02-10

**Authors:** Jozef Zustin

**Affiliations:** Orthopaedic Pathology Unit, University Medical Center Hamburg-Eppendorf, Martinistr. 52, DE-20246Hamburgj.zustin@uke.de

*Sir*—I appreciate Dr. Mereddy's comments regarding our article (Zustin and Winter 2009). In response, I would first like to discuss the issue of periprosthetic fractures following total hip resurfacing arthroplasty (THRA) somewhat more generally, and then comment on those issues with reference to the presented case.

Periprosthetic fractures following THRA can be either atraumatic, or more rarely, caused by sufficient trauma. Atraumatic fractures occur typically during the first few months after implantation and the bone hidden inside the femoral component can be either viable or display theoretically diverse pathological changes (e.g. osteonecrosis, renal osteodystrophy, tumor). Therefore, we suggested that if the femoral remnant was viable and did not display any pathological changes that might have caused its weakening, then biomechanical factors played a substantial role in the pathogenesis of those fractures. Therefore, these fractures were defined as mechanical fractures (Zustin et al. 2010b). Interestingly, the biopsy showed either no reaction to fracture (acute mechanical fracture) or findings characteristic of fracture callus or pseudoarthrosis (chronic mechanical fracture). Both fracture types are most probably associated with mechanical insufficiency of the proximal femur caused by an inaccurate surgical technique or accumulation of compressive damage (Long et al. 2009).

Currently, there is no doubt about the causative role of osteonecrosis (ON) in the pathogenesis of periprosthetic fractures following THRA (Little et al. 2005, Campbell et al. 2006, Steffen et al. 2010, Zustin et al. 2010b, c). Given the fact that the femoral head contains viable bone tissue prior to implantation, the absence of a vital reaction at the bone- (cement) -implant interface is regarded sufficient for the diagnosis of postoperative osteonecrosis and I am not aware of any data contrary to this concept, published by Bogoch et al. (1982). Nonetheless, Little et al. (2005) analyzed intraoperative biopsies from resurfaced femoral heads and found evidence of postoperative ON in 12 of 13 failures of THRA caused by fracture. Furthermore, the incidence of postnecrotic fractures was related to the extent of the ON lesion (Zustin et al. 2010a). Because healing cannot proceed within dead bone, it was not possible to further differentiate between acute or chronic postnecrotic fractures. It is true that fibrous membrane or pseudoarthrosis-like morphological changes can occur particular in cases with a collapsed ON lesion, and if we radiographically observed the pedestal sign (Madhu et al. 2010), migration and radiolucent lines in such cases, we placed them preferentially in the group of failures caused by loosening of the femoral component (Zustin et al. 2010a). Pathological fractures caused by other potential bone disease (renal osteodystrophy, tumor) have not yet been reported.

Although ON was suggested to be causative in most periprosthetic fractures in the Hamburg THRA retrieval study (2003–2009), it is noteworthy that we observed substantial changes in the failure pattern when we compared those fractures with the retrieved hips analyzed in our laboratory at the end of the study. More specifically, in retrieved femoral specimens from the latter period of time, periprosthetic fractures were less frequent and the specimens displayed smaller ON lesions. This may possibly be explained by the better surgical technique.

Traumatic fractures seem to be somewhat different from the former type because, theoretically, the femoral components are both well-seated and well-fixed during implantation, and, despite the presence of viable good quality bone remnant under the femoral component, the fractures are caused by sufficient trauma. Furthermore, notching and/or a thick cement mantle or other findings linked with inaccurate surgical technique do not seem to play any substantial role in the pathogenesis of these fractures. This complication can occur even at late follow-up and the majority of the fractures are located sub-capitally, transcervically or even in the inter- or subtrochanteric portion of the femur. When compared with the published cadaver studies (Angli et al. 2007, Richards et al. 2008, Vail et al. 2008), traumatic fractures showed some similar features (vertical fracture line in the subcapital location, possible location of the fracture line in the trochanteric area or even distally to it, ultimate load failure in experiment versus trauma).

In the presented case, we did not recognize any radiographic signs of post-implantation notching (Figure). Also, despite the sufficient time (11 weeks) between the surgical therapy of the periprosthetic fracture and the revision surgery, we did not find any cellular reaction at the bone-metal interface on the surface of the screws. In contrast to this finding, a vital reaction was apparent at the bone-cement interface of the femoral component. Moreover, although the bone tissue proximal to the fracture line was areactive and multifocally fragmented due to the collapse of necrotic bone trabeculae adjacent to the fracture line, mineralized callus formation was apparent distally to the fracture line consistent with a vital reaction to the multifocal fractured and collapsed bone. Therefore I strongly believe that in our case, the femoral remnant was viable after the implantation of the THRA but not following the periprosthetic fracture caused by the motorcycle accident and its surgical therapy.

**Figure:** Postoperative radiograph of the presented patient after implantation of the THRA.

Because traumatic fractures are quite rare, I am looking forward to seeing the results of morphological retrieval analyses by other colleagues and encourage the sharing of knowledge regarding the pathogenetic mechanisms of THRA complications.

## References

[CIT0001] Anglin C, Masri BA, Tonetti J, Hodgson AJ, Greidanus NV (2007). Hip resurfacing femoral neck fracture influenced by valgus placement. Clin Orthop.

[CIT0002] Bogoch ER, Fornasier VL, Capello WN (1982). The femoral head remnant in resurfacing arthroplasty. Clin Orthop.

[CIT0003] Campbell P, Beaule PE, Ebramzadeh E, LeDuff M, De Smet K, Lu Z (2006). The John Charnley Award: a study of implant failure in metal-on-metal surface arthroplasties. Clin Orthop.

[CIT0004] Liporace F, Gaines R, Collinge C, Haidukewych GJ (2008). Results of internal fixation of Pauwels type-3 vertical femoral neck fractures. J Bone Joint Surg (Am).

[CIT0005] Little CP, Ruiz AL, Harding IJ, McLardy-Smith P, Gundle R, Murray DW (2005). Osteonecrosis in retrieved femoral heads after failed resurfacing arthroplasty of the hip. J Bone Joint Surg (Br).

[CIT0006] Long JP, Santner TJ, Bartel DL (2009). Hip resurfacing increases bone strains associated with short-term femoral neck fracture. J Orthop Res.

[CIT0007] Madhu TS, Akula MR, Raman RN, Sharma HK, Johnson VG (2010). The Birmingham hip resurfacing prosthesis. An independent single surgeon's experience at 7-year follow-up. J Arthroplasty.

[CIT0008] Richards CJ, Giannitsios D, Huk OL, Zukor DJ, Steffen T, Antoniou J (2008). Risk of periprosthetic femoral neck fracture after hip resurfacing arthroplasty: valgus compared with anatomic alignment. A biomechanical and clinical analysis. J Bone Joint Surg (Am) (Suppl 3).

[CIT0009] Steffen RT, Athanasou NA, Gill HS, Murray DW (2010). Avascular necrosis associated with fracture of the femoral neck after hip resurfacing: histological assessment of femoral bone from retrieval specimens. J Bone Joint Surg (Br).

[CIT0010] Vail TP, Glisson RR, Dominguez DE, Kitaoka K, Ottaviano D (2008). Position of hip resurfacing component affects strain and resistance to fracture in the femoral neck. J Bone Joint Surg Am.

[CIT0011] Zustin J, Winter E (2009). Failed internal fixation due to osteonecrosis following traumatic periprosthetic fracture after hip resurfacing arthroplasty. Acta Orthop.

[CIT0012] Zustin J, Hahn M, Morlock MM, Ruther W, Amling M, Sauter G (2010a). Femoral component loosening after hip resurfacing arthroplasty. Skeletal Radiol.

[CIT0013] Zustin J, Sauter G, Morlock MM, Ruther W, Amling M (2010b). Association of osteonecrosis and failure of hip resurfacing arthroplasty. Clin Orthop.

[CIT0014] Zustin J, Krause M, Breer S, Hahn M, von Domarus C, Rüther W, Sauter G, Morlock MM, Amling M (2010c). Morphologic analysis of periprosthetic fractures after hip resurfacing arthroplasty. J Bone Joint Surg (Am).
